# In Vitro Bioactivities of Commonly Consumed Cereal, Vegetable, and Legume Seeds as Related to Their Bioactive Components: An Untargeted Metabolomics Approach Using UHPLC–QTOF-MS^2^

**DOI:** 10.3390/antiox12081501

**Published:** 2023-07-27

**Authors:** Simon Okomo Aloo, Fred Kwame Ofosu, Mary Njeri Muchiri, Selvakumar Vijayalakshmi, Choi-Geun Pyo, Deog-Hwan Oh

**Affiliations:** 1 Department of Food Science and Biotechnology, College of Agriculture and Life Sciences, Kangwon National University, Chuncheon 24341, Gangwon-do, Republic of Korea; simonaloo96@kangwon.ac.kr (S.O.A.); ofosufk17@kangwon.ac.kr (F.K.O.); vijiselva10@kangwon.ac.kr (S.V.); 2 Department of Food Science and Nutrition, School of Agriculture and Biotechnology, Karatina University, Nyeri 1957-10101, Kenya; mmuchiri@karu.ac.ke; 3 Centre of Molecular Medicine and Diagnostics (COMManD), Department of Biochemistry, Saveetha Dental College & Hospitals, Saveetha Institute of Medical and Technical Sciences, Saveetha University, Chennai 600077, India; 4 Department of Barista and Bakery, Gangwon State University, Gangneung 25425, Gangwon, Republic of Korea; kunpyochoi@hanmail.net

**Keywords:** alfalfa, broccoli, buckwheat, functional property, red cabbage, UHPLC–QTOF-MS/MS2 metabolite profiling

## Abstract

We conducted a comprehensive evaluation of the antioxidant, anti-obesity, anti-diabetic, and anti-glycation activities associated with the consumption of broccoli, red cabbage, alfalfa, and buckwheat seeds. Additionally, we explored the relationship between these biological activities and the profiles of amino acids, polyphenols, and organic acids identified in the seeds. Our findings demonstrated that red cabbage, broccoli, and buckwheat extracts exhibited significantly higher antioxidant potential compared to the alfalfa extract. Moreover, buckwheat displayed the most significant capacity for inhibiting alpha-glucosidase. Remarkably, broccoli and red cabbage demonstrated substantial anti-glycation and lipase inhibitory potentials. We identified the presence of amino acids, polyphenols, and organic acids in the extracts through untargeted metabolomics analysis. Correlation analysis revealed that pyroglutamic acid positively correlated with all the investigated functional properties. Most polyphenols made positive contributions to the functional properties, with the exception of ferulic acid, which displayed a negative correlation with all tested biological activities. Furthermore, gluconic acid and arabinonic acid among the organic acids identified displayed a positive correlation with all the functional properties. These results strongly support the anti-diabetic, anti-obesity, and anti-glycation potential of red cabbage, broccoli, and buckwheat seeds.

## 1. Introduction

Since ancient times, humans have relied on cereal-, vegetable-, and legume-based products as their main dietary staples. There has been a growing trend in consuming foods derived from cereals, vegetables, and legumes, as they are believed to possess various health-promoting benefits. *Brassicaceae* vegetables have been cultivated worldwide for many decades, and one of the remarkable features of this botanical family is the presence of diverse metabolites that offer significant health advantages. Glucosinolates, along with their metabolites, such as isothiocyanates and indoles, are the most extensively studied class of bioactive compounds in *Brassicaceae* [1]. Additionally, this plant family contains other bioactive compounds, including phenolic compounds, phytoalexins, and phytosteroids, which, though important, have received less attention in research [2]. Six species of the Brassica genus, namely, *Brassica juncea*, *Brassica rapa*, *Brassica napus*, *Brassica carinata*, *Brassica oleracea*, and *Brassica nigra*, are widely distributed across the globe [3]. Among these, *Brassica oleracea* encompasses major vegetable species such as broccoli, cauliflower, cabbage, kale, kohlrabi, and brussels sprouts [3]. The consumption of these vegetables has been associated with several health benefits, including anti-cancer effects [4] and anti-diabetic properties [5]. On the other hand, various cereals are cultivated globally and utilized to produce cereal-based foods. Buckwheat, a widely grown cereal in the *Polygonaceae* family, is particularly notable. The *Polygonaceae* family comprises approximately 1200 plant species. Buckwheat seeds are highly nutritious and have been found to possess a wide array of beneficial effects. They are primarily consumed as dehulled seeds (raw groats) in breakfast cereals [6]. Moreover, due to being gluten-free, buckwheat seeds are commonly milled into flour and used to prepare bakery products such as bread, cookies, and noodles [6]. Buckwheat grains are especially rich in polyphenols, including catechins, rutin, and quercetin [7,8]. They also contain higher amounts of amino acids such as lysine, glutamic acid, proline, arginine, and aspartic acid than cereal proteins [9]. The diverse metabolites present in buckwheat have been reported to exhibit various biological activities, including cholesterol-lowering effects and anti-obesity properties [9].

In addition to vegetables and cereals, legume consumption plays a significant role in the human diet due to its associated health benefits. Legumes are excellent sources of protein, micronutrients, and several phytochemicals [10]. They are known to contain all the essential amino acids, making them valuable components of a balanced diet when consumed in combination with cereals [10]. Alfalfa (*Medicago sativa*), belonging to the legume family *Fabaceae*, has been extensively cultivated for many years, primarily as animal feed, due to its mineral, fiber, and protein content. Alfalfa has been found to contain numerous polyphenols, including quercetin, naringenin, myricetin, kaempferol, apigenin, daidzein, and genistein [11,12]. Alfalfa leaf has been long considered as a potential protein source to meet human global nutritional needs, although its use in dietary formulations is limited due to its poor nutritional quality resulting from anti-nutritional factors (particularly saponins) and poor sensory attributes (bitterness and darkness) [13]. Nevertheless, studies have supported the therapeutic roles of alfalfa in humans, particularly their antioxidant, antimicrobial, anti-inflammatory, and anti-cancer activities [11,12]. Currently, researchers and food processors are keen on exploring the health benefits of plant-based foods as related to the bioactive compounds they contain. Polyphenols are one of the major bioactive compounds existing in vegetables such as red cabbage, where cyanidin (73.6–117.7 mg/kg) has been designated as the predominant flavonoid and the main contributor to the antioxidant properties [14]. Buckwheat seed’s rutin (518.54–1447.87 mg/100 g of DW) is the principal compound responsible for its antioxidant activity [15], while alfalfa seed has been characterized with high polyphenol (51.68 gallic acid equivalent/g) and flavonoid (18.55 quercetin equivalent/gram) contents, which account for its potent antioxidant and anti-inflammatory effects [16]. Thus, the bioactive compounds in vegetables, cereal, and legumes may differ highly, varying their bioactivities. Our study aimed to compare the health benefits and metabolite profiles of commonly consumed vegetables (broccoli and red cabbage), cereal (buckwheat), and legumes (alfalfa). We also sought to evaluate the correlation between the bioactive components (amino acid, polyphenol, and organic acid profiles) and the functional properties of the samples tested.

## 2. Materials and Methodology

### 2.1. Plant Materials Collection

**Figure 1:**
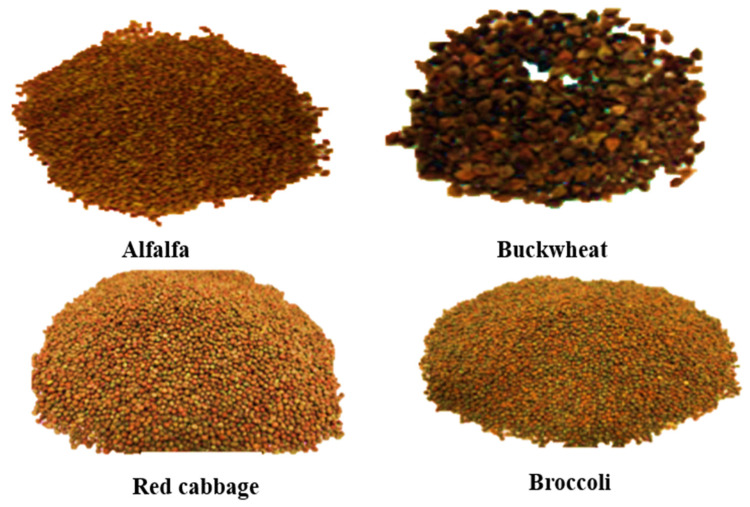
Representatives of legume, cereal, and vegetable seeds used in the study. All the samples (alfalfa, buckwheat, red cabbage, and broccoli seeds) were kindly provided by Charm-Chae-One, Ltd. (Jincheon, Chungbuk, Republic of Korea).

Alfalfa, buckwheat, red cabbage, and broccoli seeds were obtained from Charm-Chae-One, Ltd. (Jincheon, Chungbuk, Republic of Korea). The samples were dried, pulverized into a fine powder, and stored in a cold room throughout the study period. The representative images of the samples are shown in Figure 1.

### 2.2. Chemicals

The 2, 2-diphenyl-1-picrylhydrazyl (DPPH) and the 2, 2-azino-bis (3-ethylbenzothiazoline-6-sulfonic acid (ABTS) were purchased from Sigma-Aldrich (Seoul, Republic of Korea). The reagents, including enzymes, substrates (4-nitrophenyl α-d-glucopyranoside), and standards (orlistat, acarbose, and trolox), were all obtained from Sigma-Aldrich (Seoul, Republic of Korea). All other reagents were of the analytical grade.

### 2.3. Preparation of Ethanol Extract

All the samples were ground and weighed into 70% ethanol in a 1:20 (weight/volume) ratio each. The mixture was extracted following the procedure described in our previous article [7]. The final supernatant was concentrated under a vacuum, freeze-dried, and then used for further analysis by reconstituting in 70% ethanol.

### 2.4. Total Phenolic, Flavonoids, and Saponin Contents

Total phenolic content (TPC), total flavonoid content (TFC), and total saponin content (TSC) of ethanol extracts were measured based on sample absorbance using spectra Max i3 plate reader (Molecular Devices Korea, LLC, Seoul, Republic of Korea). The TPC and TFC analyses were based on our previous study [7]. The TPC and TFC were determined by measuring the absorbance at 765 nm and 510 nm, respectively. The TPC was expressed in milligrams per 100 g of ferulic acid equivalent, dry weight (mg/100 g FAE, DW), while TFC was expressed in milligrams per 100 g catechin equivalent dry weight (mg/100 g CE, DW) of the sample. Total saponin was conducted by mixing 100 µL of the extract with 1 mL of 72% sulfuric acid and then adding 100 µL of 8% vanillin in ethanol. The mixture was incubated at 60 °C for 20 min, and the absorbance was measured at 544 nm. The TSC expressed as milligram soysaponin B equivalents per 100 g of sample (mg/100 g SSBE, DW). All the experiments were performed in triplicates.

### 2.5. DPPH and ABTS Radical Inhibition Assays

The 2, 2-diphenyl-1-picrylhydrazyl (DPPH) scavenging activity was performed by mixing the extracts (1 mg/mL) or standard (trolox) with freshly prepared DPPH radical solution. The absorbance of the mixture was measured at 517 nm. 2, 2-azino-bis (3-ethylbenzothiazoline-6-sulfonic acid (ABTS) radical was prepared by mixing 5 mL of 2.45 mM Potassium persulfate with 5 mL of 7 mM ABTS solution and then incubating the mixture for 16 h in the dark. The ABTS working solution was then prepared by diluting 2 mL ABTS* with 200 mL ethanol to reach an absorbance of 0.70 at 734 nm. An amount of 100 µL of ABTS working solution was mixed with 1.0 mL of the sample or standard at different concentrations and incubated for 30 min before reading the absorbance at 734 nm using a microplate reader against a blank. The experiment was performed in triplicate. The percentage DPPH and ABTS inhibition capacities were calculated using the formula below:

$$\mathrm{Inhibition}\;(\%)=\left(\frac{O.D\;control-O.D\;test\;sample}{O.D\;control}\right)\times 100,$$

where *OD control* is the absorbance for the control blank (negative control), and *OD test sample* is the absorbance for the tested extracts or standard (Trolox).

### 2.6. Pancreatic Lipase Inhibition Assay

The pancreatic lipase inhibition assay was conducted following the protocol described in the literature [7] using 50 µL (1 mg/mL in 70% ethano) of the extracts mixed with 50 µL (50 U/mL) of lipase enzyme in methyl cellosolve at room temperature. The mixture was preincubated for 10 min at room temperature before adding 100 µL of 1 mM 4-Methylumbelliferyl (4-MU) in methyl cellosolve and then further incubated for 30 min at room temperature. Finally, 100 µL of 0.1 M, pH 4.2 of sodium citrate solution was used to stop the reaction. The fluorescence of the tested samples was measured using a microplate reader at wavelengths 355 nm and 460 nm. The experiment was performed in triplicates and the percentage lipase inhibition capacity was calculated as follows:

$$\mathrm{Lipase}\;\mathrm{Inhibition}\;(\%)=[1-\left(\frac{Ftest-Ftest\;blank}{\;Fcontrol-Fcontrol\;Blank}\right)]\times 100,$$

where *Ftest* and *Ftest blank* represent the fluorescent readings for the test samples with and without the substrate 4-Methylumbelliferyl (4-MU) oleate, respectively, while Fcontrol and Fcontrol blank were the fluorescent readings of control with and without the substrate 4-MU oleate, respectively.

### 2.7. Alpha-Glucosidase Inhibitory Assay

The α-glucosidase inhibitory activity was measured using 1 mg/mL of plant extracts following the procedure by [17]. Acarbose at the same concentration was used as the positive control. Briefly, the extract was prepared in 10 mM potassium phosphate buffer (pH 6.8). An amount of 100 µL extract was pipetted into the 24-well plate, and 100 µL of the freshly prepared alpha-glucosidase enzyme (0.5 U/mL) was added, followed by 300 µL of 10 mM potassium phosphate buffer (pH 6.8). The mixture was allowed to settle for 15 min, pre-incubation time at 37 °C. After the 15 min pre-incubation, 100 µL of the substrate (5 mM p-nitrophenol-α-D-glucopyranoside) was added. The mixture was incubated at 37 °C for a further 15 min. An amount of 400 µL of 200 mM sodium carbonate was used to stop the reaction before absorbance reading was taken at 405 nm using a SpectraMax i3 plate reader (Molecular Devices Korea, LLC, Seoul, Republic of Korea). The sample blanks containing test samples, substrate, and buffer without α-glucosidase were also assayed. The control test sample contained all other reagents except the extract, which was replaced by 70% ethanol. The experiment was performed in triplicate. The percentage inhibition (%) of α-glucosidase of the test sample or standard was calculated using the formula below:

$$\mathrm{Inhibition}\;(\%)=\left(\frac{AC-AS}{AC}\right)\times 100,$$

where *AC* and *AS* are the absorbances recorded for the control and test samples, respectively.

### 2.8. Inhibition of AGEs Formation

AGE inhibition assay was performed by mixing (333 µL) of bovine serum albumin (BSA, 5.0 mg/mL) mixed with an equal volume of D-glucose (36 mg/mL) and the test samples or aminoguanidine (concentrations 1.0 mg/mL). The solutes were dissolved in 0.2 M phosphate buffer saline (pH 7.4) containing sodium azide (0.02% *w*/*v*). The mixture was incubated at 37 °C for a week and fluorescent monitored using a microplate reader using 340 and 420 nm as the excitation and emission wavelengths, respectively. The experiment was conducted in duplicate, and the percentage of the AGE inhibition was obtained as follows:

$$\mathrm{AGE}\;\mathrm{formation}\;\mathrm{inhibition}\;(\%)=\left[1-\left(\frac{Fluorescent\;of\;the\;test}{Fluorescent\;of\;control}\right)\right]\times 100.$$

### 2.9. UHPLC–QTOF-MS/MS^2^ Metabolite Identification

The ultra-high performance liquid chromatography–quadrupole time-of-flight mass spectrometry (UHPLC–QTOF-MS^2^) hybrid technique is an emerging approach in the chromatographic analysis that has been successfully employed for the fast and high resolution analysis of compounds in plant extracts and other origins with required sensitivity. The technology supports structure elucidation and identification of fragmentation patterns of different compounds. The LC–MS/MS analysis was performed using a UHPLC (SCIEX ExionLC AD system, Framingham, MA, USA) according to the experiment previously performed in our lab [18]. The UHPLC system was connected to a controller, an autosampler, column oven, a pump, a degasser, and a photodiode array detector (ExionLC) coupled to a quadrupole time-of-flight mass spectrometer (QTOF-MS) (X500R QTOF). The analytical column used was a 100 × 3 mm, Accucore C18 column (Thermo Fisher Scientific, Waltham, MA, USA). Solvent A was a mixture of water with 0.1% formic acid and solvent B was methanol. The chromatographic analysis was conducted at a 0.4 mL/min flow rate. A linear gradient was programmed for 25 min as follows: 0–3.81 min, 9% to 14% B; 3.81–4.85 min, 14% to 15% B; 4.85–5.89 min, 15% B; 5.89–8.32 min, 15% to 17% B; 8.32–9.71 min, 17% to 19% B; 9.71–10.40 min, 19% B; 10.40–12.48 min, 19% to 26% B; 12.48–13.17 min, 26% to 28% B; 13.17–14.21 min, 28% to 35% B; 14.21–15.95 min, 35% to 40% B; 15.95–16.64 min, 40% to 48% B; 16.64–18.37 min, 48% to 53% B; 18.37–22.53 min, 53% to 70% B; 22.53–22.88 min, 70% to 9% B; and 22.88–25.00 min, 9% B. The injection volume was 5 µL. The QTOF-MS was set for the negative mode through a mass range of 100–1000 and a resolution of 5000. The capillary and cone voltages used to record full mass spectra were 1.45 kV and 30 V, respectively. The flow rate of Helium (the cone gas) was 45 L/h, while the flow rate of the desolvation gas (N_2_) was 900 L/h. The temperature of N_2_ was 250 °C, the ion source temperature was 120 °C, while the collision energies needed to obtain the MS/MS spectra were set at 15, 20, and 30 V.

### 2.10. Statistical Analysis

The analytical procedures were performed using GraphPad Prism 8.0 (GraphPad Software, San Diego, CA, USA). The mean values of the various activities of extracts were determined using a one-way analysis of variance (ANOVA) and Tukey’s test at a *p < 0.05* significance level. The analysis results were presented as mean ± standard deviation (S.D). All the experiments were conducted in triplicate. The principal component analysis (PCA) and heat map were performed using OriginPro 2021 and ClustVis (https://biit.cs.ut.ee/clustvis. Last accessed on 15 May 2023). Correlation analysis was conducted using OriginPro 2022.

## 3. Results

### 3.1. Total Phenolic (TPC), Flavonoid (TFC), Saponin (TSC) Contents

**Figure 2:**
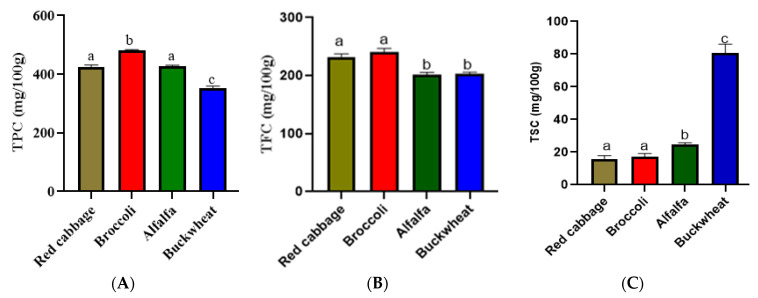
Quantitative analysis of phytochemicals in ethanolic extracts of alfalfa, red cabbage, broccoli, and buckwheat seeds. (**A**) The total polyphenol content (TPC), (**B**) total flavonoid content (TFC), and (**C**) total spaonin content (TSC) of the tested extracts. Significant differences among values were marked with the different letters in each category (*p* < 0.05). All the experiments were performed in triplicates.

The total polyphenol content of the extracts determined by the Folin–Ciocalteau method is reported in Figure 2A. The TPC in alfalfa, buckwheat, broccoli, and red cabbage seed extracts ranged between 351.55–481.75 mg/100 g FAE. The total flavonoid content was determined using aluminium trichloride (AlCl3) and shown in Figure 2B. The TFC varied from 210.434-240.357 mg/100 g CE). Total saponin content (TSC) was determined using 72% sulfuric acid and 8% vanillin dissolved in ethanol. The highest saponin content was found in buckwheat (80.78 mg/100 g SSBE), while red cabbage had the least amount (15.82 53 mg/100 g SSBE) (Figure 2 C).

### 3.2. In Vitro Antioxidant, AGEs Formation Inhibition, Alpha-Glucosidase Inhibition, and Pancreatic Lipase Inhibition Activities

**Figure 3:**
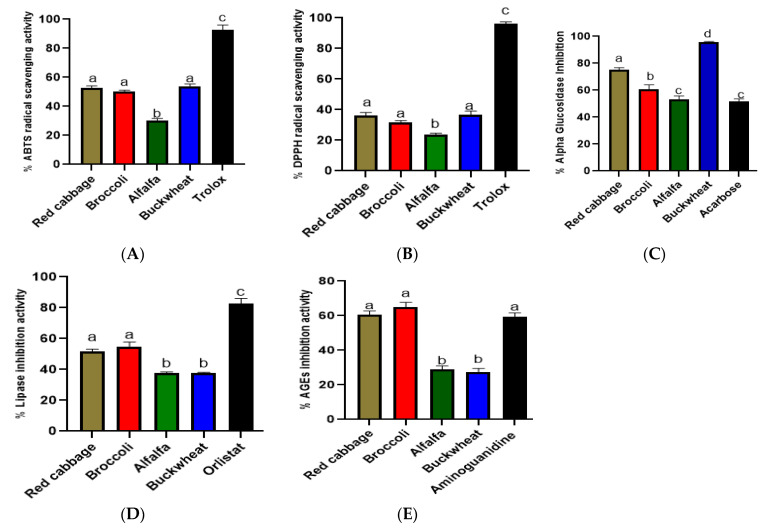
The functional properties of ethanolic extracts of alfalfa, red cabbage, broccoli, and buckwheat seeds. (**A**,**B**) The antioxidant, (**C**) alpha-glucosidase, (**D**) lipase inhibition, and (**E**) AGEs inhibition capacities of the tested extracts. There were significant differences among values marked with different letters in each category (*p* < 0.05). All the experiments were performed in triplicates.

Metabolic diseases are characterized by excessive production of reactive oxygen species, which is closely related to oxidative stress. One of the aims of this study was to find out the antioxidant activity of red cabbage, broccoli, alfalfa, and buckwheat seeds. For in vitro antioxidant assays, 2, 2-diphenyl-1-picrylhydrazyl (DPPH) and 2, 2-azino-bis (3-ethylbenzothiazoline-6-sulfonic acid (ABTS) as free radical agents were made to react with the 70% ethanol extracts of alfalfa, buckwheat, red cabbage, and broccoli seeds. The most significant ABTS scavenging potential was observed in vegetable and cereal extracts. When expressed in percentage, red cabbage showed 52.71%; broccoli, 50.26%; buckwheat, 53.85%; and alfalfa, 30.29% ABTS scavenging potential. DPPH scavenging activity of the extracts followed a similar trend as ABTS but with relatively lower values. Red cabbage, broccoli, alfalfa, and buckwheat exerted 36.29%, 31.97%, 23.64%, and 35.59% DPPH inhibition ability, respectively. These results are shown in Figure 3A,B.

The current research also evaluated the abilities of red cabbage, broccoli, alfalfa, and buckwheat to inhibit α-glucosidase and pancreatic lipase. The results of these tests are illustrated in Figure 3C,D. The alpha-glucosidase inhibition ranged from 53.05% to 75.35%, and the lipase inhibition from 37.56% to 54.99%. Red cabbage (57.4%) and broccoli (54.99%) demonstrated the strongest potential to inhibit lipase activity in vitro. In comparison, buckwheat seed extract possessed the best alpha-glucosidase inhibition capacity (95.5%), followed by red cabbage seed extract (75.35%). Alfalfa seed displayed the weakest lipase (37.56%), which was not significantly difference from alpha-glucosidase (53.05%) inhibition capacities among the extracts.

Since advanced glycation end products (AGEs) increase obesity, oxidative stress, diabetes, and inflammation, natural products which can prevent the formation of these products appear to be beneficial in health [19]. In Figure 3E, broccoli (64.92%) exerted the highest potential to inhibit AGEs formation, which was not significantly different from red cabbage values (60.52%). Alfalfa (28.89%) and buckwheat (27.59%) seeds exhibited lower AGEs formation inhibition values, indicating that vegetable extracts were more anti-AGEs than cereal and legume extracts.

### 3.3. Comprehensive Profiling of Possible Bioactive Compounds in Alfalfa, Buckwheat, Broccoli, and Red Cabbage Extracts Using UHPLC–QTOF-MS/MS^2^

In the present study, UHPLC–QTOF-MS analysis allowed for the comprehensive characterization of the metabolites in alfalfa, buckwheat, broccoli, and red cabbage extracts via an untargeted approach. The identification strategy of the detected metabolites was based on their corresponding retention times (RT), experimental *m*/*z*, molecular formulas, and MS^2^ fragments. The central focus for employing UHPLC–QTOF-MS/MS was to detect the amino acid, polyphenols, and profiles of organic acids in the tested extracts. Thus, using this approach, we identified 15 amino acids (Peaks 1 to 15), 9 polyphenols (Peaks 16 to 25), and 8 organic acids (Peaks 26 to 33) from data generated by UHPLC–QTOF-MS analysis in the extracts (Table 1). Thus, metabolomics based on UHPLC–QTOF-MS/MS^2^ was effective for the analysis of constituents in the extracts. In addition to the amino acids, polyphenols, and organic acids described above, we performed comprehensive profiling of other possible bioactive compounds in red cabbage, broccoli, alfalfa, and buckwheat seeds. The metabolites (peaks, 34–53) were identified based on the above-stated procedure. Important bioactive compounds, including glucosinolates and alkaloids, were extensively characterized and listed along with their retention time (RT), *m*/*z* fragment pattern, molecular mass, and literature evidence as shown in Table 1.

### 3.4. Heatmap Visualization and Principal Component Analysis of Tentatively Identified Metabolites

**Figure 4:**
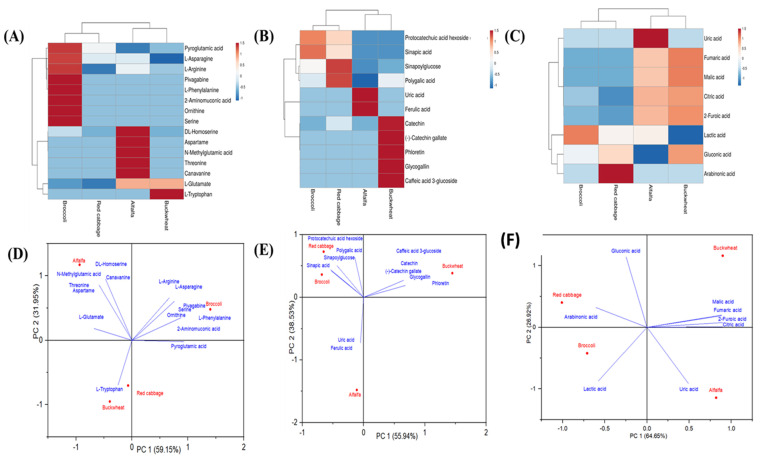
Heatmaps and PC bi-plots based on the identified metabolites in alfalfa, red cabbage, broccoli, and buckwheat extracts. Heatmaps developed for (**A**) polyphenols, (**B**) amino acids, and (**C**) organic acids identified in the exracts; PC bi-plots for (**D**) polyphenols, (**E**) amino acids, and (**F**) organic acids identified in the extracts. The heatmap colors range from blue to red. The red color of the heatmap shows high concentration (denoted by peak area) of the particular metabolites, while blue color demonstrate low concentration.

The diversity and content of the identified amino acids, polyphenols, and organic acids in the four samples were visualized by using a heatmap. Further, the principal component analysis was used to discriminate the extracts based on the levels of the tentatively identified amino acids, polyphenols, and organic acids. The heatmap visualization and bi-plot charts for PCA are shown in Figure 4. The scale range from blue to red in the heatmap (Figure 4A–C). The red colour means that the corresponding compound is high in the given extract, whereas blue indicates a low metabolite level in the sample.

### 3.5. Exploring the Contribution of Amino Acids, Polyphenols, and Organic Acids to the Functional Properties of Red Cabbage, Broccoli, Alfalfa and Buckwheat

**Figure 5:**
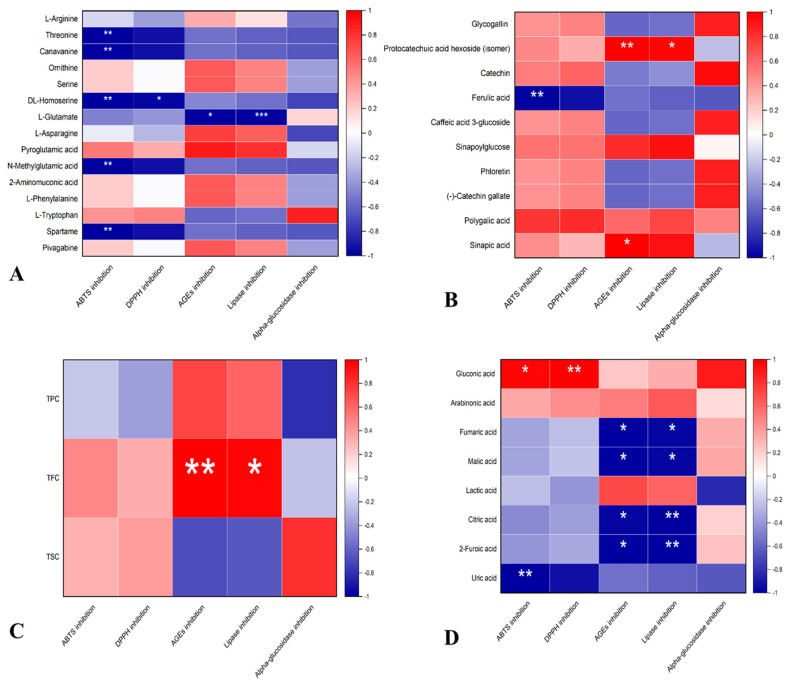
Heatmaps for correlation of amino acids (**A**), individual polyphenols (**B**), TPC, TFC, and TSC (**C**), and organic acids (**D**) with DPPH, ABTS, alpha-glucosidase inhibition, pancreatic lipase inhibition, and AGEs formation inhibition. The red color denotes positive correlation, while blue color indicates negative correlation. The * *p* < 0.05, ** *p* < 0.01, *** *p* < 0.001 represent significance of the correlation.

The UHPLC–QTOF-MS/MS^2^ findings demonstrated that red cabbage, broccoli, alfalfa, and buckwheat extracts are associated with amino acids, polyphenols, and organic acids. Previous studies have shown that amino acids, polyphenols, and organic acids compounds from plant materials contribute to the health benefits of vegetables, legumes, and cereals foods [37,38]. Therefore, our objective was to perform a correlation analysis to establish the possible contributions of amino acids, polyphenols, and organic acids in the tested functional properties of red cabbage, broccoli, and alfalfa seeds. The outcome of correlation analysis performed by OriginPro statistical software is described by heatmaps in Figure 5.

### 3.6. Correlation between Glucosinolates Identified in Red Cabbage and Broccoli Seeds and Functional Properties

**Figure 6:**
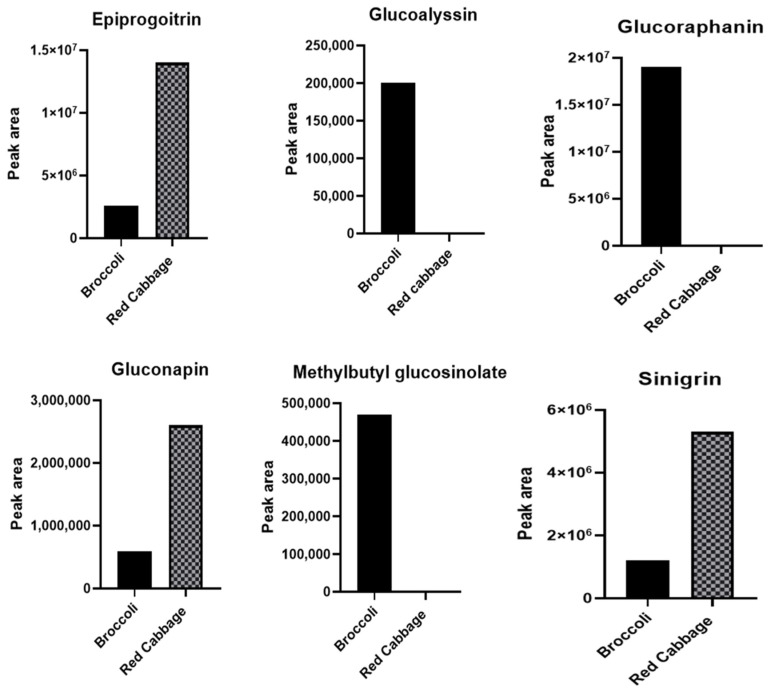
Relative peak areas of the UHPLC–QTOF-MS^2^ identified glucosinolates in red cabbage and broccoli seed ethanol extracts.

Glucosinolates have been identified as the primary bioactive secondary compounds found in *Brassica.* They have been associated with a variety of health benefits, including antioxidant [39], anti-cancer [4], and anti-diabetic [5] activities. The variation of these compounds in Brassica vegetables affects their intake and health-promoting properties. In this study, we identified six glucosinolates using UHPLC–QTOF-MS/MS^2^. The UHPLC–QTOF-MS/MS^2^-btained chromatograms of the identified glucosinolates (in Table 1) are shown in Figure S1. The corresponding peak areas of the glucosinolates identified in the current study were plotted as shown in Figure 6.

**Figure 7:**
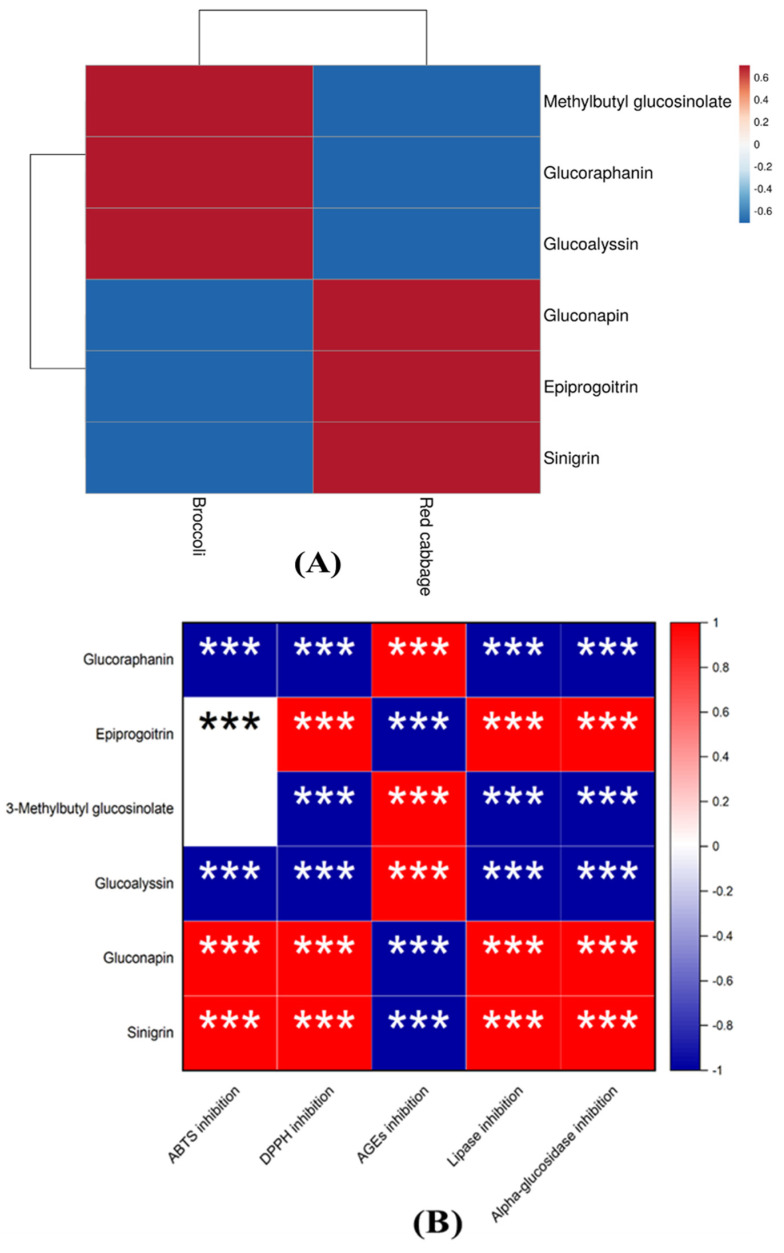
Heatmaps showing the levels of glucosinolates in the ethanolic extracts of red cabbage and broccoli ethanol extracts (**A**) and their correlation with antioxidant, lipase inhibition, and alpha-glucosidase inhibition (**B**). The red color denotes positive correlation, while blue color indicates negative correlation. The *** *p* < 0.001 represent significance of the correlation.

Furthermore, because of their reported positive impact in the health benefits, we conducted a correlation analysis between the identified glucosinolates and the tested health-related studies: antioxidant potential, anti-AGEs formation ability, lipase inhibition, and alpha-glucosidase inhibition capacities of red cabbage and broccoli seeds. Figure 7A presents a heatmap visualization showing the relative content of the identified glucosinolates in red cabbage and broccoli seeds. Additionally, Figure 7B illustrates how these compounds are correlated with the biological activities we tested. According to Figure 7A, gluconapin, epiprogoitrin, and sinigrin were higher in red cabbage than in broccoli seeds. The remaining glucosinolates were higher in broccoli seed (indicated by red colour). Moreover, all the identified glucosinolates positively correlated with the health activities, revealing their potent contribution to the antioxidant potential, anti-AGEs inhibition ability, lipase inhibition, and alpha-glucosidase inhibition capacities of red cabbage and broccoli seeds (Figure 7B).

## 4. Discussion

Polyphenols and flavonoids are abundant in plants and are widely recognized as excellent antioxidants that can inhibit formation of free radicals in the body [38]. On the contrary, saponins found in certain plants are classified as antinutrients; nevertheless, they still have potentially positive effects on health [40]. Among the seeds tested, broccoli seeds exhibited the highest total polyphenol content at 481.75 mg/100 g FAE, whereas buckwheat seeds had the lowest content at 351.55 mg/100 g FAE. Red cabbage and alfalfa seeds displayed 424.15 mg/100 g FAE and 427.85 mg/100 g FAE, DW of TPC (total polyphenol content), respectively (Figure 2A). It is worth noting that our findings differ from a previous study, which reported a higher amount of phenolic compounds expressed in milligrams of gallic acid equivalent per gram of dry weight in red cabbage (18.51) compared to broccoli (6.39) [41]. Similar results were also reported by Şengül, Yildiz, and Kava, who observed a significantly higher TPC in raw red cabbage (350.94 μg GAE/mg of sample) than broccoli (154.94 ± 3.30 μg GAE/mg of sample) [42]. On the other hand, in our study, we observed no substantial difference in flavonoid content between red cabbage and broccoli (231.95 vs. 240.357 mg/100 g CE) (Figure 2B). Broccoli was reported to contain TFC of 316 µg quercetin/g, fresh weight [43]; red cabbage, 108.19 mg CE, DW [14]; buckwheat, 0.67–2.25 mg/g [44]; while alfalfa seed contains 18.55 mg quercetin equivalent/gram of these compounds [16]. Therefore, the vegetables, cereal, and legumes studied could vary highly in their total polyphenol and flavonoid content. Moreover, the total saponin content varied considerably among the vegetable, legume, and cereal samples. Buckwheat had the highest concentration of saponins at 80.78 mg/100 g SSBE, followed by alfalfa (24.53 mg/100 g SSBE), broccoli (17.0453 mg/100 g SSBE), and red cabbage (15.8253 mg/100 g SSBE) (Figure 2C), respectively (Figure 2C). A previous study indicated the presence of significant amounts of saponins in buckwheat seeds [45] and alfalfa seeds [46], which limits their utilization due to the negative effects of these compounds in food.

The primary line of defense against the development of metabolic disorders involves the inhibition of oxidative stress and certain hydrolyzing enzymes. The present findings strongly align with previous research, which stated that broccoli and red cabbage seeds exhibit potential as antioxidants [24,47,48]. As shown in Figure 3A,B, the extracts showed some antioxidant ability, and alfalfa displayed the weakest potential to inhibit the radicals. In Figure 3C,D, all the tested seeds displayed the ability to inhibit pancreatic lipase and alpha-glucosidase enzymes. For alpha-glucosidase, the highest and lowest inhibition were recorded for buckwheat and alfalfa, respectively. For pancreatic lipase enzyme, broccoli seed extracts demonstrated the highest inhibition capacity, which did not differ significantly from red cabbage values. Alfalfa and buckwheat displayed the lowest inhibition capacity against lipase enzyme, revealing lower anti-obesity effects compared to red cabbage or broccoli. Alpha-glucosidase and pancreatic lipase are the pivotal enzymes responsible for carbohydrate and triglyceride hydrolysis, respectively, and could significantly impact blood glucose and triglyceride levels, respectively [49]. Consequently, inhibiting the activities of α-glucosidase and pancreatic lipase has been identified as an effective approach for managing diabetes and obesity. Plant-based inhibitors of α-glucosidase and pancreatic lipase have been shown to effectively inhibit the hydrolysis of carbohydrates and fats, which may lead to reduced release of sugar and triglycerides into the bloodstream and delayed absorption in the intestinal tract. A study indicated the ability of processed buckwheat to inhibit free radicals and α-glucosidase enzyme [50]. Red cabbage (Koda variety) also reportedly demonstrated inhibitory activity against α-glucosidase (IC_50_ = 3.87 ± 0.12 mg, DW of cabbage/mL) and lipase (IC_50_ = 1.57 ± 0.06 mg, DW/mL), as well as antioxidant capacity in ABTS (trolox equivalent antioxidant capacity (TEAC) = 141 ± 4.71 μmol/g DW) [51]. Thus, together with the current investigation, studies have proved vegetable, cereal, and legume seeds as antioxidant, anti-obesity, and anti-diabetic ingredients.

Obesity and diabetes are complex metabolic disorders characterized by accumulation of deleterious changes in cells and tissues and progressive deterioration of structural integrity and physiological function across multiple organ systems. Advanced glycation end products (AGEs) are a heterogeneous group of macromolecules that are formed by the nonenzymatic glycation of proteins, lipids, and nucleic acids. AGEs are among the major groups of macromolecules affecting the structural integrity and physiological function of organs, and they are being suggested as diabetes biomarkers [19]. Preventing the formation of these compounds is crucial in obesity and diabetes management as they are regarded as driving factors for the severity of these conditions. Studies have reported that alfalfa, buckwheat, broccoli, and red cabbage have the potential to prevent the formation of AGEs [7,52,53,54]. Reports have also demonstrated the ability of buckwheat seed extracts-enhanced wheat bread to display antiglycation effects [55]. We substantiated these findings further by demonstrating that these seeds possess a significant effect against AGEs formation (Figure 3E). Broccoli and red cabbage possessed the highest inhibitory activities against AGEs formation. Their potent anti-AGEs did not significantly differ from those observed for the standard drug, aminoguanidine (Figure 3E).

Metabolomics is an ‘omics’ approach that can comprehensively identify metabolites in a biological sample. This method holds great potential for directly elucidating plant metabolites and metabolic processes. One of the most current metabolomic techniques used for plant compound identification is UHPLC–QTOF-MS/MS2-based metabolite profiling. Analyzing the extracts using this technique allowed for the identification of various amino acids, polyphenols, organic acids, and other compounds in the samples under study. The following amino acids were tentatively identified: L-Arginine (Peak **1**) and threonine (Peak **2**), by comparing spectral data obtained using UHPLC–QTOF-MS/MS2 with relevant literature evidence [20,21]. Peak **3**, exhibiting 86, 118, and 130 ion fragments patterns, was tentatively identified as canavanine by comparing spectral data with METLIN data and cross-checking with literature reports [22]. Bence et al. demonstrated that canavanine, an L-arginine analogue, primarily exists in leguminous plants [22], thus confirming our present finding that this compound was present in alfalfa seeds (a legume) but absent in red cabbage, broccoli, and buckwheat seeds. Ornithine (peak **4**), serine (peak **5**), DL-Homoserine (peak **6**), L-Glutamate (peak **7**), L-Asparagine (peak **8**), pyroglutamic acid (peak **9**), N-Methylglutamic acid (peak **10**), 2-Aminomuconic acid (peak **11**), L-Phenylalanine (peak **12**), L-Tryptophan (peak **13**), aspartame (peak **14**), and pivagabine (peak **15**) were tentatively identified by comparing their exact molecular mass, molecular formula, and ion fragment pattern against the METLIN database and cross-checking with literature evidence, as shown in Table 1. Moreover, the compound with the elemental composition C_9_H_17_NO_3_ has rarely been reported in the literature. Bunning and fellow researchers reportedly identified this compound in two ageing twins but labeled it as unknown [56]. In their recent work, Zhang et al. characterized a compound with the same molecular mass and formula in *Zanthoxylum bungeanum* pericarp extract and identified it as pivagabine [26]. Hence, based on the similarity in molecular mass and the formula with Zhang and colleagues’ data, the compound C_9_H_17_NO_3_ was identified as pivagabine, which is a hydrophobic 4-aminobutyric acid. As a conclusion of amino acid detection, ornithine, serine, 2-Aminomuconic acid, L-Phenylalanine, and pivagabine were found in broccoli seeds but were absent in red cabbage, alfalfa, and buckwheat. Threonine and aspartame were found in alfalfa seeds, while L-Tryptophan was present in buckwheat.

In Table 1, phenolic compounds were widely detected in the extracts (Peak **16–25**). Glycogallin (peak **16**) displayed a [M − H]^−^ at *m/z* 331.07 and underwent fragmentation at *m/z* 168 (representing the deprotonated aglycon) due to the loss of the hexose moiety. Its identification was based on spectral information and relevant literature evidence [27]. Peak **17,** with a [M − H]^−^ of 315.07, was identified as protocatechuic acid hexoside by comparing its fragment ions with those reported in the literature [28]. Peak **18** produced fragment ions at 245, 203, 151, 137, and 109, consistent with those reported by Wang et al. [28] leading to its identification as catechin. Peak **19,** with a parent ion [M – H]^−^ at *m/z* 193.05, was recognized as ferulic acid based on the characteristic fragment ions at *m/z* 193 and 151 [29]. According to UHPLC–QTOF-MS/MS analysis, peak **20** (Retention time of 11.09 min) generated a [M – H]^−^ at *m/z* 341.09 and exhibited a characteristic ion fragment pattern at *m/z* 179 [M – H-162]^−^ corresponding to caffeic acid, resulting from the cleavage of glucose residues. Other ions observed for this compound were found at *m/z* 161 [caffeic acid-H-H_2_O]^−^ and 135 [caffeic acid-H-CO_2_]^−^, in line with previous studies [30]. Thus, the compound was identified as caffeic acid 3-glucoside. Peak **21** yielded a deprotonated molecule at *m/z* 385.11, along with fragment ions matching those reported by Wang et al. [28] at 223 [M-C_6_H_10_O_5_-H]^−^, 208 [M-C_6_H_10_O_5_-CH_3_- H]^−^, 179 [M-C_6_H_10_O_5_-CO_2_-H]^−^, 164 [M-C_6_H_10_O_5_-CO_2_-CH_3_-H]^−^, and 149 [M-C_6_H_10_O_5_-CO_2_-C_2_H_6_-H]^−^. Therefore, the compound was identified as sinapoylglucose, a phenolic antioxidant compound previously described by Wang and colleagues [28]. Peak **22** exhibited an abundant [M − H]^−^ precursor ion at *m/z* 273.08, along with product ions at *m/z* 255, 149, and 137, corresponding to phloretin according to reports by Maciejewska-Turska and Zgórka [31]. Peaks **23**, **24**, and **25,** with *m/z* values of 2441.08, 488.31, and 223.06, respectively, were identified as (-)-catechin gallate, polygalic acid, and sinapic acid by comparing ion fragment patterns, exact molecular masses, and compound formulas with literature evidence [24,32] and METLIN spectral data. Our study has indicated that catechin and catechin gallate are abundant in buckwheat seeds, consistent with previous findings [9].

Organic acids play a crucial role as secondary metabolites in plants. In this study, eight organic acids were identified in the 70% ethanol extract of red cabbage, broccoli, alfalfa, and buckwheat (Table 1). The identification of these compounds involved a combination of the methods described earlier. Peaks **26**, **27**, **28, 29, 30, 31, 32**, and **33** were tentatively identified as gluconic acid, arabinonic acid, fumaric acid, malic acid, uric acid, lactic acid, citric acid, and 2-Furoic acid, respectively. Arabinonic acid was particularly abundant in red cabbage seeds, while fumaric and malic acids were found high in alfalfa and buckwheat seeds. Gluconic acid was not detected in the alfalfa extract, whereas lactic acid was present in all the extracts except the buckwheat seed extract. The composition of organic acids in the samples under investigation has received limited attention. However, it has been suggested that organic acids are important plant compounds that may serve as vital antioxidant metabolites [57]. Moreover, apart from the identified compounds described above (amino acids, polyphenols, and organic acids), the UHPLC–QTOF-MS/MS2 analysis identified peak **34–53** as some bioactive metabolites in the tested seeds (Table 1). Peak **34** was identified as nicotianamine by comparing its ion fragment pattern to METLIN spectral data and cross-referencing with appropriate literature evidence [33]. Nicotianamine is abundant in graminaceous plants and acts as a precursor of phytosiderophores, which are essential compounds for acquiring iron from the soil. Peaks **35, 51**, and **52** produced parent ions [M − H]^−^ at *m/z* 200, 495, and 485, respectively. These compounds were identified as alkaloids: camalexin, capparidisine, and quinadoline. Their identification was confirmed by examining their exact mass and molecular formula and comparing them with METLIN spectral data and/or appropriate literature evidence as shown in Table 1. It has been reported that camalexin and glucosinolates actively participate in defense against abiotic factors in certain plants [25]. Six glucosinolates (glucoraphanin, epiprogoitrin, sinigrin, glucoalyssin, 2 or 3-methylbutyl glucosinolate, and gluconapin) were identified in red cabbage and broccoli seeds. The compound with the elemental formula C_12_H_23_NO_9_S_2_ (RT, 6.14) displayed a fragment pattern at *m/z* 74, 95, 96, 259, and 388, corresponding to the suggested compounds, either 2 or 3-methylbutyl glucosinolate (further elucidation is needed for exact identification). All the glucosinolates were identified by comparing their fragment patterns with appropriate literature data [35].

Heatmap visualization and principal component analysis (PCA) were conducted to effectively differentiate the tested samples based on their polyphenols, amino acids, and organic acid contents. As depicted in Figure 4A, among the 15 amino acids analyzed, tryptophan was found to be the most abundant in buckwheat. N-Methylglutamic acid, DL-Homoserine, aspartame, threonine, and L-Canavanine were the highest in alfalfa seeds. Pyroglutamic acid, L-Asparagine, L-Arginine, L-Phenylalanine, 2-Aminomuconic acid, ornithine, and serine were abundant in broccoli seeds, while red cabbage exhibited moderate levels of most of the identified amino acids. Regarding polyphenols, buckwheat displayed the highest levels of caffeic acid 3-glucoside, glycogallin, phloretin, catechin, and (-)-Catechin gallate. Ferulic acid was the most abundant in alfalfa, while red cabbage contained higher amounts of polygalic acid and sinapoylglucose. Prominent polyphenols in broccoli seeds were protocatechuic acid hexoside and sinapic acid (Figure 4B). Among the identified organic acids, five out of seven were predominantly present in buckwheat seeds (Figure 4C). Furthermore, PCA was performed on amino acids, polyphenols, and organic acids to assess the differences and similarities between red cabbage, broccoli, alfalfa, and buckwheat. The two-dimensional PCA plot (Figure 4D) revealed distinct groupings of amino acids based on their levels in each sample. The first two principal components (PC1 and PC2) accounted for 59.15% and 31.95% of the total variance, respectively, displaying an overall variance contribution rate of 91.1% for amino acids (Figure 4D). Notably, broccoli and alfalfa seeds exhibited a higher abundance of amino acids compared to red cabbage and buckwheat. Similarly, the PC1 and PC2 plots derived from multivariate analysis of polyphenols indicated that buckwheat had the highest number of identified polyphenols, followed by broccoli, while red cabbage did not show a significant difference (Figure 4E). Figure 4F further supported these findings, indicating that buckwheat seeds had a distinct profile of organic acids.

To illustrate the influence of polyphenols, amino acids, and organic acids on the functional properties of the samples under investigation, we conducted correlation analysis using OriginPro 2022. Figure 5A presents the results of the correlation analysis with amino acids. Pyroglutamic acid exhibited a relatively strong positive correlation with ABTS (r = 0.574) and a moderate positive correlation with DPPH (r = 0.304). Ornithine, serine, and L-tryptophan displayed positive correlations with both ABTS and DPPH scavenging capacities. Conversely, threonine, DL-Homoserine, canavanine, N-Methylglutamic acid, and aspartame exhibited a strong negative correlation with antioxidant capacity. Among the amino acids, pyroglutamic acid demonstrated the strongest positive correlation with AGEs inhibition (r = 0.881) and lipase inhibition (r = 0.907). Additionally, although not as strong as pyroglutamic acid, 2-Aminomuconic acid and L-Phenylalanine, ornithine, serine, L-Asparagine, and pivagabine also displayed positive correlations with AGEs inhibition and lipase inhibition. On the other hand, L-glutamate exhibited the strongest negative correlation with AGEs inhibition (r = −0.987) and lipase inhibition (r = −0.976), followed by N-Methylglutamic acid, L-Tryptophan, aspartame, canavanine, and threonine, respectively. Regarding alpha-glucosidase inhibition capacities, only L-Tryptophan showed a strong positive correlation (r = 0.87). At the same time, L-glutamate displayed only a weak positive correlation, and the remaining amino acids exhibited a negative correlation with alpha-glucosidase inhibition activity. In addition to their nutritional functions, amino acids are vital compounds within plants that may have potential biological effects. Thus, in the current investigation, the correlation analysis depicted pyroglutamic acid as one of the most essential amino acids, exhibiting a positive correlation with almost all the investigated functional properties (Figure 5A). Interestingly, some studies have indicated anti-diabetic properties of pyroglutamic acid in a rat model [58]. Moreover, certain amino acids such as phenylalanine and tryptophan play an important role in immune system function [59], while some have been reported as antioxidants. On the hand, the individual phenolic compounds present in the extracts of red cabbage, broccoli, alfalfa, and buckwheat exhibited distinct correlation patterns with antioxidant, anti-obesity, anti-diabetic, and AGEs inhibition activities (Figure 5B). Most of these polyphenols showed a positive correlation with antioxidant capacities, despite the negative correlation between total polyphenols and ABTS and DPPH scavenging capacities. Protocatechuic acid hexoside, sinapoylglucose, and sinapic acid displayed the strongest positive correlation with AGEs formation inhibition and pancreatic lipase inhibition capacities of the extracts. Glycogallin, catechin, and (-)-Catechin gallate were strongly positively correlated with alpha-glucosidase inhibition. These findings suggest that the abundance of glycogallin, catechin, and (-)-Catechin gallate in buckwheat played a crucial role in its high alpha-glucosidase inhibition observed in Figure 3C. Notably, ferulic acid negatively correlated with all the tested biological activities. In Figure 5C, total flavonoid content (TFC) exhibited a strong positive correlation with lipase inhibition (r = 0.999) and AGEs inhibition (r = 0.995), which were all significant compared to total phenolic content (TPC). From these experiments, it can be concluded that ferulic acid, uric acid, and polygalic acid did not significantly contribute to the tested health benefits of red cabbage, broccoli, alfalfa, and buckwheat seeds. On the other hand, other individual polyphenols played a significant role in exerting health benefits, even though total polyphenol content did not (except in anti-AGEs formation and lipase inhibition activities).

All organic acids, except gluconic acid and arabinonic acid, exhibited a negative correlation with the antioxidant values of the extract (Figure 5D). Gluconic acid displayed a stronger positive correlation with antioxidant values than arabinonic acid. While almost all the organic acids showed a negative correlation with antioxidant activity, uric acid and lactic acid were the only ones that exhibited a negative correlation with alpha-glucosidase. Moreover, gluconic acid, lactic acid, and arabinonic acid were positively correlated with AGEs inhibition and lipase inhibition values. Interestingly, gluconic acid and arabinonic acid demonstrated a positive correlation with all the functional properties, suggesting that these compounds may have a greater potency in exerting health-beneficial effects compared to other organic acids identified. 

Finally, since the 1970s, extensive research has been conducted on glucosinolates and their metabolites, exploring their advantageous and detrimental impacts on human and animal nutrition. These compounds have been identified as contributing factors to the anti-cancer and antioxidant properties of Brassica vegetables [60]. They also contribute to the distinctive taste of *Brassica* vegetables. In Figure 7B, the analysis showed that all the glucosinolates could potentially significantly affect all the tested functional properties of broccoli and red cabbage seeds. Thus, going by the conclusion from other studies [1,60] and the current findings, we speculate that the health effects associated with the consumption of *Brassica* vegetables are primarily attributed to the presence of glucosinolates.

## 5. Conclusions

In this study, we evaluated the functional properties (antioxidant, anti-obesity, anti-diabetic, and anti-glycation activities) of commonly consumed vegetable (broccoli and red cabbage), legume (alfalfa), and cereal (buckwheat) seeds and the correlation between these biological activities and metabolite profiles. Red cabbage, broccoli, and buckwheat demonstrated a higher antioxidant potential than alfalfa. Buckwheat extracts exhibited the highest alpha-glucosidase inhibition capacity among all the studied samples. At the same time, broccoli and red cabbage showed higher AGEs formation and lipase inhibitory potentials than alfalfa and buckwheat. Untargeted metabolomics using UHPLC–QTOF-MS/MS confirmed the presence of amino acids, polyphenols, and organic acids in the extracts. The majority of the identified polyphenols and amino acids positively correlated with the functional activity tested. Among the organic acids, gluconic acid and arabinonic acid displayed a positive correlation with all the functional properties studied. Further study is needed to verify the in vivo health benefits of red cabbage, broccoli, alfalfa, and buckwheat seeds.

## Figures and Tables

**Table 1:** List of UHPLC–QTOF-MS/MS^2^-identified compounds in the alfalfa, buckwheat, broccoli, and red cabbage seed extracts. The compounds have been listed along with their retention time (RT), [M − H]^−^, molecular formula, peak areas, and fragment patterns.

Peak No.	Metabolites	RT Per Min	[M − H]^−^ (*m*/*z*)	Molecular Formula	Alfalfa	Buckwheat	Broccoli	Red Cabbage	Fragment Pattern	Reference
**Amino acids**										
1	L-Arginine	0.7	173.10	C_6_H_14_N_4_O_2_	77,000	65,000	110,000	52,000	173	[20]
2	Threonine	0.69	118.05	C_4_H_9_NO_3_	190,000	0.00	0.00	0.00	56, 74	[21]
3	Canavanine	0.7	175.08	C_5_H_12_N_4_O_3_	310,000	0.00	0.00	0.00	86, 118, 130	[22]
4	Ornithine	0.71	131.08	C_5_H_12_N_2_O_2_	0.00	0.00	63,000	0.00	131, 88	[23]
5	Serine	0.77	104.04	C3H_7_NO_3_	0.00	0.00	29,000	0.00	44, 60	[21]
6	DL-Homoserine	0.81	118.05	C_4_H_9_NO_3_	190,000	0.00	29,000	0.00	74, 100	[24]
7	L-Glutamate	0.82	146.04	C_5_H_9_NO_4_	220,000	220,000	24,000	0.00	102, 146	[23]
8	L-Asparagine	0.78	131.05	C_4_H_8_N_2_O_3_	54,000.00	0.00	130,000	54,000	58, 113	[23]
9	Pyroglutamic acid	1.05	128.04	C_5_H_7_NO_3_	48,000	69,000	160,000	100,000	289, 72	METLIN database
10	N-Methylglutamic acid	1.18	160.06	C_6_H_11_NO_4_	45,000	0.00	0.00	0.00	58, 142	METLIN database
11	2-Aminomuconic acid	1.21	240.05	C_6_H_7_N_7_O_4_	0.00	0.00	27,000	0.00	128	METLIN databases
12	L-Phenylalanine	2.98	164.07	C_9_H_11_NO_2_	0.00	0.00	72,000	0.00	77, 103	[23]
13	L-Tryptophan	5.9	203.08	C_11_H_12_N_2_O_2_	0.00	68,000	0.00	0.00	205, 188, 118, 91, 116, 142	[23,25]
14	Aspartame	8.99	293.11	C_14_H_18_N_2_O_5_	470,000	0.00	0.00	0.00	-	METLIN databases
15	Pivagabine	15.34	186.11	C_9_H_17_NO_3_	0.00	0.00	78,000	0.00		[26]
**Polyphenols**										
16	Glycogallin	2.48	331.07	C_13_H_16_O_10_	0.00	53,000	0.00	0.00	168	[27]
17	Protocatechuic acid hexoside (isomer)	4.03	315.07	C_13_H_16_O_9_	0.00	0.00	130,000	100,000	163, 152	[28]
18	Catechin	9.68	289.07	C_15_H_14_O_6_	0.00	350,000	0.00	68,000	245, 203, 151, 137, 109	[28]
19	Ferulic acid	10.89	193.05	C_10_H_10_O_4_	79,000	0.00	0.00	0.00	193, 151	[29]
20	Caffeic acid 3-glucoside	11.09	341.09	C_15_H_18_O_9_	0.00	190,000	0.00	0.00	179, 281, 221, 161, 135	[30]
21	Sinapoylglucose	13.66	385.11	C_17_H_22_O_10_	0.00	120,000	620,000	1,300,000	223, 208, 179 164	[28]
22	Phloretin	15.99	273.08	C_15_H_14_O_5_	0.00	50,000	0.00	0.00	255, 149 137	[31]
23	(-)-Catechin gallate	16.24	441.08	C_22_H_18_O_10_	0.00	310,000	0.00	0.00	125, 124, 145, 303	[24]
24	Polygalic acid	16.67	487.31	C_29_H_44_O_6_	250,000	330,000	320,000	420,000	61, 174, 239	METLIN database
25	Sinapic acid	16.75	223.061	C_11_H_12_O_5_	0.00	0.00	110,000	75,000	121, 149, 164, 179, 223, 193, 208	[32]
**Organic acid**										
26	Gluconic acid	0.83	195.05	C_6_H_12_O_7_	0.00	640,000	370,000	540,000	177, 159, 129	[27]
27	Arabinonic acid	0.85	165.04	C_5_H_10_O_6_	0.00	0.00	0.00	180,000	-	-
28	Fumaric acid	1.04	115.00	C_4_H_4_O_4_	96,000	120,000	0.00	0.00	-	-
29	Malic acid	1.05	134.02	C_4_H_6_O_5_	300,000	380,000	0.00	0.00	71, 133, 59	METLIN database
30	Uric acid	1.21	168.03	C_5_H_4_N_4_O_3_	150,000	0.00	0.00	0.00	168	METLIN database
31	Lactic acid	1.21	89.02	C_3_H_6_O_3_	45,000	0.00	70,000	43,000	89	METLIN database
32	Citric acid	1.22	191.02	C_6_H_8_O_7_	770,000	790,000	570,000	510,000	173,155, 131, 129. 127, 111 191	[30]
33	2-Furoic acid	1.23	111.01	C_5_H_4_O_3_	250,000	260,000	180,000	170,000	65, 70	[24]
**Other compounds**										
34	Nicotianamine	0.76	303.14	C_12_H_21_N_3_O_6_	0.00	0.00	0.00	24,000	305, 287, 241, 169, 114	[33]
35	Camalexin	0.77	200.04	C_11_H_8_N_2_S	0.00	0.00	45,000	0.00	201, 160, 142, 59	[25]
36	Dihydrouracil	0.77	114.04	C_4_H_6_N_2_O_2_	0.00	0.00	48,000	0.00	111, 112, 113	[34]
37	Glucoraphanin	1.02	436.04	C_12_H_23_NO_10_S_3_	0.00	0.00	19,000,000	0.00	291, 275, 259, 195, 97	[35]
38	Epiprogoitrin	1.08	388.04	C_11_H_19_NO_10_S_2_	0.00	0.00	2,600,000	14,000,000	96, 74, 95, 274, 90, 135,259, 149	[35]
39	Robinose	1.09	326.12	C_12_H_22_O_10_	0.00	100,000	0.00	0.00	-	-
40	Sinigrin	1.12	358.03	C_10_H_17_NO_9_S_2_	0.00	0.00	1,200,000	5,300,000	74, 95, 96, 274	[35]
41	Uridine	1.22	243.06	C_9_H_12_N_2_O_6_	100,000	0.00	0.00	0.00	110, 82, 122, 66	
42	Glucoalyssin	1.21	450.06	C_13_H_25_NO_10_S_3_	0.00	0.00	200,000	0.00	96, 95, 192, 165, 256, 386	[35]
43	Adenosine	1.23	268.10	C_10_H_13_N_5_O_4_	560,000	370,000	0.00	0.00	136, 268	
44	Gluconapin	1.9	372.04	C_11_H_19_NO_9_S_2_	0.00	0.00	590,000	2,600,000	74, 96, 119, 174, 274, 372	[35]
45	4-Sulfophthalic anhydride	2.31	227.97	C_8_H_4_O_6_S	920	0.00	2600	5000	-	-
46	2-Benzoylmalononitrile	2.93	170.05	C_10_H_6_N_2_O	0.00	0.00	0.00	290,000	-	-
47	2 or 3-Methylbutyl glucosinolate	6.14	388.07	C_12_H_23_NO_9_S_2_	0.00	0.00	470,000	0.00	74, 95, 96, 259, 388	[35]
48	Emiglitate	10.42	355.16	C_17_H_25_NO_7_	0.00	0.00	2,400,000	2,300,000	93, 119,147, 355	METLIN database
49	Actinonin	13.33	384.25	C_19_H_35_N_3_O_5_	0.00	420,000	380,000	380,000	111, 180, 224	[24]
50	7-Caffeoylsedoheptulose	14.88	372.11	C_16_H_20_O_10_	0.00	400,000	0.00	0.00	77, 121, 175, 249	METLIN database
51	Capparidisine	16.11	495.24	C_27_H_33_N_3_O_6_	0.00	0.00	830,000	0.00	-	-
52	Quinadoline A	16.27	485.21	C_27_H_27_N_5_O_4_	0.00	0.00	56,000	0.00	-	-
53	1-Hexadecylamine	17.00	242.29	C_16_H_35_N	490,000	540,000	0.00	0.00	136, 268	[36]

## Data Availability

Data is contained within the article and supplementary material.

## Supplementary Materials

The following supporting information can be downloaded at: https://www.mdpi.com/2076-3921/12/8/1501/s1, Figure S1: UHPLC-QTOF-MS/MS2 extracted ion fragments of glucosinolates that were detected in red cabbage and broccoli seed extracts.

## Abbreviations

ABTS	2, 2-azino-bis (3-ethylbenzothiazoline-6-sulfonic acid
AGE	Advanced glycation end produs
CE	Catechin equivalent
FAE	Ferrulic acid equivalent
SSBE	Soysaponin B equivalents
DPPH	2-diphenyl-1-picrylhydrazyl
OD	Optical density
TPC	Total phenolic content
TFC	Total flavonoid content
TSC	Total saponin content
4-MU	4-Methylumbelliferyl
