# Supplementary material for: In Vitro Bioactivities of Commonly Consumed Cereal, Vegetable, and Legume Seeds as Related to Their Bioactive Components: An Untargeted Metabolomics Approach Using UHPLC–QTOF-MS2
Source: Antioxidants (Basel). 2023 Jul 27;12(8):1501. doi: 10.3390/antiox12081501 (PMC10451260; doi:10.3390/antiox12081501)
Supplement: Supplementary file 1 [file antioxidants-12-01501-s001.zip › antioxidants-2496656-supplementary.pdf]

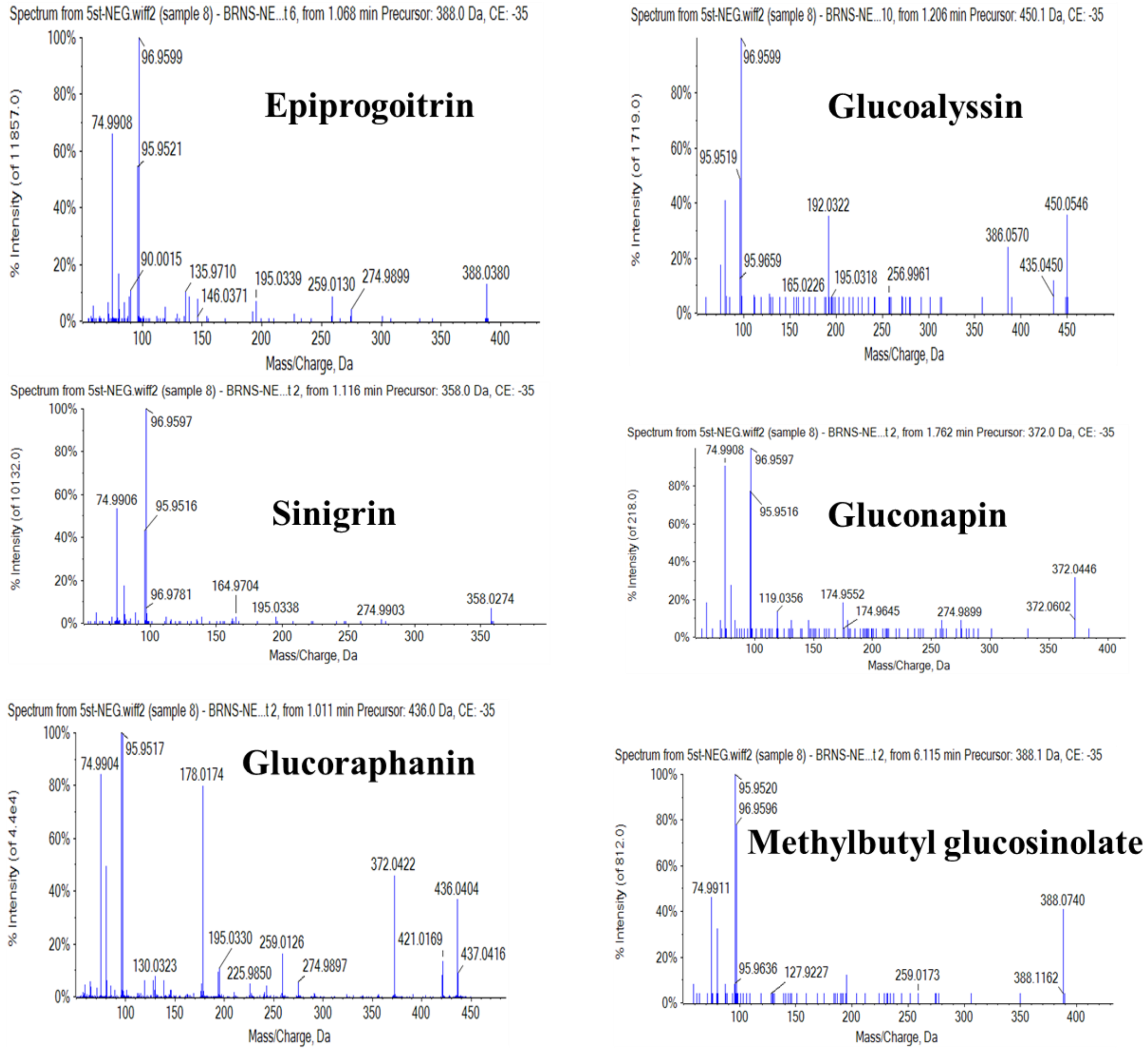

Figure S1. UHPLC-Q-TOF-MS/MS² extracted ion fragments of glucosinolates that were detected in red cabbage and broccoli seed extracts
